# A multimodal approach identifies lactate as a central feature of right ventricular failure that is detectable in human plasma

**DOI:** 10.3389/fmed.2024.1387195

**Published:** 2024-09-12

**Authors:** Anna Hemnes, Niki Fortune, Katie Simon, Irina A. Trenary, Sheila Shay, Eric Austin, Jamey D. Young, Evan Britain, James West, Megha Talati

**Affiliations:** ^1^Division of Allergy, Pulmonary, and Critical Care Medicine, Department of Medicine, Vanderbilt University Medical Center, Nashville, TN, United States; ^2^Department of Chemical and Biomolecular Engineering, Vanderbilt University, Nashville, TN, United States; ^3^Department of Pediatrics, Vanderbilt University Medical Center, Nashville, TN, United States; ^4^Department of Molecular Physiology and Biophysics, Vanderbilt University, Nashville, TN, United States; ^5^Department of Cardiology, Vanderbilt University Medical Center, Nashville, TN, United States

**Keywords:** pulmonary arterial hypertension, BMPR2 mutation, right ventricular dysfunction and lipotoxicity, multi-omics, metabolic pathways, H9c2 cultured cardiomyocyte

## Abstract

**Background:**

In PAH metabolic abnormalities in multiple pathways are well-recognized features of right ventricular dysfunction, however, prior work has focused mainly on the use of a single “omic” modality to describe a single deranged pathway. We integrated metabolomic and epigenomic data using transcriptomics in failing and non-failing RVs from a rodent model to provide novel mechanistic insight and translated these findings to accessible human specimens by correlation with plasma from PAH patients.

**Methods:**

Study was conducted in a doxycycline-inducible BMPR2 mutant mouse model of RV failure. Plasma was collected from controls and PAH patients. Transcriptomic and metabolomic analyses were done on mouse RV tissue and human plasma. For mouse RV, we layered metabolomic and transcriptomic data for multiple metabolic pathways and compared our findings with metabolomic and transcriptomic data obtained for human plasma. We confirmed our key findings in cultured cardiomyocyte cells with BMPR2 mutation.

**Results:**

In failing mouse RVs, (1) in the glycolysis pathway, glucose is converted to lactate via aerobic glycolysis, but may also be utilized for glycogen, fatty acid, and nucleic acid synthesis, (2) in the fatty acid pathway, FAs are accumulated in the cytoplasm because the transfer of FAs to mitochondria is reduced, however, the ß-oxidation pathway is likely to be functional. (3) the TCA cycle is altered at multiple checkpoints and accumulates citrate, and the glutaminolysis pathway is not activated. In PAH patients, plasma metabolic and transcriptomic data indicated that unlike in the failing BMPR2 mutant RV, expression of genes and metabolites measured for the glycolysis pathway, FA pathway, TCA cycle, and glutaminolysis pathway were increased. Lactate was the only metabolite that was increased both in RV and circulation. We confirmed using a stable isotope of lactate that cultured cardiomyocytes with mutant BMPR2 show a modest increase in endogenous lactate, suggesting a possibility of an increase in lactate production by cardiomyocytes in failing BMPR2 mutant RV.

**Conclusion:**

In the failing RV with mutant BMPR2, lactate is produced by RV cardiomyocytes and may be secreted out, thereby increasing lactate in circulation. Lactate can potentially serve as a marker of RV dysfunction in PAH, which warrants investigation.

## Introduction

Pulmonary arterial hypertension (PAH) is a rare fatal disease characterized by progressive remodeling of the pulmonary vasculature causing an increase in PA pressure that leads to right ventricular (RV) dysfunction and ultimately failure. In PAH, animal and human data have pointed to metabolic abnormalities in multiple pathways as features of RV dysfunction, often investigating individual pathways in isolation. In recent years, studies have used state-of-the-art omics technology platforms to understand PAH pathogenesis (1–3) and have begun to show the complexity of metabolic derangement in the PAH RV. However, single-omics studies generally cannot fully elucidate the systems-level biological process in the PAH RV.

Integration of multi-omics data may allow for a complete understanding of biological systems (4–7) and their complex crosstalk. In a rodent model of PH, a multi-omics approach has been used to study underlying molecular and metabolic pathways (8, 9). In human and animal models of PH, multi-omics studies were mainly done in plasma and lung tissue samples (10), including a recent study comparing trans-right ventricular and transpulmonary metabolite gradients in PAH (11). We also conducted a study recently in PAH patients to determine cardiac MR spectroscopy-measured RV triglyceride content and compared it to plasma metabolites analyzed using non-targeted metabolomics studies before and after metformin therapy (12). Most prior studies describing altered metabolism in PAH demonstrated a shift from oxidative phosphorylation to 0 glycolysis, glutaminolysis, and impairment in fatty acid oxidation were conducted in pulmonary endothelial and smooth muscle cells (13–20). In the RV in PAH, prior work has primarily focused on substrate preference and not studied global metabolic shifts and what underlies these changes (17, 21). So far it is not well understood whether sequential changes that occur in multiple metabolic pathways in the failing RV can potentially correlate to metabolic changes that occur in circulation and contribute to disease pathobiology.

A major challenge to not only understanding the mechanisms of RV failure but also translating this knowledge to human disease is the lack of correlation of RV molecular findings with human blood findings. Unlike biomarkers such as BNP, such mechanistic markers could point clinicians to specific deranged pathways in individual patients, offer early diagnosis of RV dysfunction, and may ultimately pave the way for a precision medicine approach to therapy of RV failure in humans with PAH. We have previously published that mice with universal expression of mutant BMPR2 (the predominant cause of heritable PAH) develop disproportionate RV failure to their pulmonary vascular disease. Thus, this model holds tremendous promise to study mechanisms of RV failure that are highly relevant to human disease—and may even be detectable in the blood of affected individuals. Further, comparison of RV findings in this model with plasma from humans with heritable PAH offers a unique ability to translate rodent findings to human disease. In this novel study, we used a multi-omics approach coupled with key functional assays to understand dysfunctional metabolic pathways in a highly relevant mouse model of RV failure in PAH with mutant BMPR2. We further sought to correlate our metabolic findings with metabolic changes in the circulation in the PAH patients. Using this approach of integrating metabolomics and transcriptomics across multiple metabolic pathways in relevant tissues, our goal is to understand whether observed changes in multiple metabolic pathways are RV tissue-specific and whether they may correlate with findings in peripheral blood of humans with heritable PAH.

## Materials and methods

### PAH patient and healthy controls information

This study was approved by the Vanderbilt University Medical Center Institutional Review Board (IRB #9401) and all subjects provided informed consent. PAH patients for this study were consecutively enrolled in the Vanderbilt Pulmonary Hypertension Research Cohort (VPHRC), a prospective institutional registry containing detailed clinical information and biological specimens collected over 30 years that have been previously described (22). PAH was defined according to contemporary guidelines. Healthy control subjects with no known cardiovascular disease and no cardiopulmonary signs or symptoms were enrolled in the VPHRC. Plasma lactic acid data was generated by mass spectrometry core at VUMC using plasma samples from PAH patients (*n* = 41) and healthy controls (*n* = 20). The metabolite data was generated as described before (23). The gene expression data was generated using NS_human_metabolism_v1.0 assay +custom panel_C7746 (Pulmonary Arterial Hypertension related genes) (Supplementary Table 1) at VUMC core using whole blood from PAH patients (*n* = 4) and healthy controls (*n* = 10).

### Mice protocol for metabolomic analysis

All animal procedures were approved by the Institutional Animal Care and Use Committee of Vanderbilt University School of Medicine. We used a mouse model of mutant Bmpr2 expression: the Rosa26-rtTA2 × TetO7-Bmpr2^*R*899*X*^ FVB/N mice previously described (24, 25), called BMPR2R899X for brevity (26), in which mutant BMPR2 is universally expressed. Expression of transgene occurs only after initiation of doxycycline. Transgene-negative mice were used as littermate controls and were administered doxycycline as well. In brief, 6-week-old mice were fed a regular diet (5% fat) for 6 weeks along with doxycycline, after which these animals underwent echocardiography, and invasive hemodynamic measurement, and then sacrificed to harvest RV tissue. BMPR2 mutant mice with low cardiac index as previously published (27) were used to study RV failure. Mouse RV tissue samples snap frozen in liquid nitrogen and stored at −80°C from control (*n* = 7) and BMPR2 mutant mice (*n* = 8). To investigate the effect of BMPR2 mutation on RV dysfunction mass spectrometry-based metabolomic analyses were done as described previously (28, 29). Briefly, RV tissue was subjected to methanol extraction and split into aliquots for analysis by ultrahigh performance liquid chromatography/mass spectrometry (UHPLC/MS) in either the positive or negative ion mode or by gas chromatography/mass spectrometry (GC/MS). Internal standards and controls for signal blank, technical replicates, and instrument performance were spiked into the samples and tracked throughout the analysis. Metabolite concentrations were determined by automated ion detection, and manual visual curation, and were analyzed by two-way ANOVA using software developed by Metabolon. The unit of measurement for all the metabolites is denoted as “Scaled intensity” by Metabolon. All bar graphs were generated using the GraphPad Prism10 software.

### Mice protocol for transcriptomics analysis using NanoString^®^ nCounter assay

BMPR2^*R*899*X*^ and control mice were fed a high-fat chow (60% lard, WD) with doxycycline beginning at 6 weeks of age and continued for 6 weeks. To investigate the effect of BMPR2 mutation on RV dysfunction NanoString^®^ nCounter Technology (NanoString Technologies, Seattle, WA, USA) assay was performed using the nCounter^®^ metabolic pathways panel as described previously (30). Total RNA was extracted from RV tissue using an RNeasy mini kit (Qiagen). 100 ng of extracted mRNA were used as input material. Subsequently, a hybridization process was conducted overnight at a temperature of 65°C, utilizing 50 bases of nCounter Reporter and Capture probes. Following the process of hybridization, the samples were subsequently introduced into the nCounter Prep Station to purify the samples and immobilize the target/probe complex onto the cartridge. The nCounter Digital Analyzer was utilized to perform a high-density scan (555 fields of view) for each assay. This scan was designed to enumerate individual fluorescent barcodes and measure the abundance of target RNA molecules in each sample. Analysis of multiplexed gene expression of 768 genes was performed following the manufacturer’s instructions on the Counter Flex system and using the nSolver software v4.0. Transcription copies were standardized using the geometric mean of 20 maintenance genes for baseline and normalization. The threshold count value of 50 was the baseline subtraction parameter; gene expression fold changes were calculated by comparing RV tissue from control mice with BMPR2 mutant mice. The *p*-values for gene expression were evaluated using raw data for differential gene expression. The unit of measurement for all the genes is denoted as “Average counts” by NanoString. All bar graphs were generated using the GraphPad Prism10.

### H9c2 cells used for cell culture experiments

H9c2 rat cardiac myoblasts were purchased from the American Type Culture Collection (ATCC, Manassas, VA, USA), and maintained in a growth medium comprising supplemented Dulbecco’s modified Eagle’s medium supplemented (DMEM), 10% fetal bovine serum (FBS), 2 mM glutamine, 1 mM pyruvate and 100 U/mL penicillin, and 100 mg/mL streptomycin, in humidified air (5% CO_2_) at 37°C. To induce the differentiation into cardiac myocytes, the H9c2 myoblasts were transferred to a differentiation medium, which was composed of DMEM, 1% FBS, 2 mM glutamine, 1 mM pyruvate, 100 U/mL penicillin, and 100 mg/mL streptomycin. Cells starved in differentiation medium for 48 h were used for experiments (31). The H9c2 cells were stably transfected with mutant BMPR2 plasmids containing a mutation in the kinase domain (Mutant: M; BMPR2 gene with a C993T mutation resulting in R332X, mutation shown to be present in HPAH families). G418 was used for the selection of positive clones (32). In addition, we generated a H9c2 stable clone containing an empty vector, which served as a control (C).

### PAS staining for glycogen in mouse RV tissue

Paraffin-embedded RV tissue slides from control and BMPR2 mutant mice were deparaffinized and rehydrated with 1X PBS. Next, these slides were incubated in 0.5% periodic acid solution for 5 min and then rinsed with distilled water. After that, the slides were placed in Schiff reagent for 15 min and then washed in lukewarm tap water for 5 min. Mayer’s hematoxylin was used as a counter stain for 1 min. The slides were washed in tap water for 5 min, dehydrated, and mounted using Xylene 60. The staining was visualized under a bright field using a Nikon light microscope. The images were analyzed using Image J, and the protocol is described briefly. Firstly, the image was converted to RGB, followed by color deconvolution of the image by selecting “H DAB” option to deconvolve. Then we adjusted the threshold for the DAB-stained image. Finally, we measured the stained area and perimeter. The reported measurement is a fold change in the number of positive pixels to the total number of pixels measured (image dimensions) in RV tissue from control mice compared to BMPR2 mutant mice.

### Enzyme activity assay for lactate dehydrogenase (LDH) in H9c2 cells and human RV tissue and isocitrate dehydrogenase (IDH) in H9c2 cells

For measuring LDH activity in H9c2 (control and mutant) cells and in RV tissue from 1] 3 control RV and 1 Dilated cardiomyopathy RV (as disease control) and 5 PAH (2 HPAH and 3 IPAH) we used lactate dehydrogenase activity colorimetric assay kit (Cat No# K726-500 BioVision, Milpitas, CA, USA). For measuring IDH activity in H9c2 cells Isocitrate Dehydrogenase activity assay kit (Cat No# MAK062 Sigma-Aldrich, St. Louis, MO, USA) was used. For both assays, the protocol was followed as per the manufacturer’s instructions.

### Western blotting in H9c2 cells

H9c2 cells were homogenized in RIPA buffer (PBS, 1% Ipegal, 0.5% sodium deoxycholate, 0.1% SDS) with proteinase and phosphatase inhibitor cocktails (Sigma-Aldrich, St. Louis, MO, USA). Protein concentration was determined by Bradford assay and was stored at −70°C until use. For western blotting, Glud1 antibody (PA5-28301 Invitrogen, ThermoFisher Scientific) and ß-actin (4967S, Cell Signaling Technologies, Danvers, MA, USA) were used as the primary antibodies, and donkey anti-rabbit (711-035-152, Jackson ImmunoResearch Laboratories, Inc., West Grove, PA, USA) was used as a secondary antibody.

### Stable isotope labeling in H9c2 cells

For isotope tracer studies, control BMPR2 mutant H9c2 cells were cultured in media containing 10 mM sodium [U-^13^C_3_]L-lactate (CLM-1579-MPT-PK; Sodium L-lactate; Cambridge Isotope Laboratories, Inc; Tewksbury, MA, USA) in place of unlabeled L-lactate for 24 h. Analytes were extracted into ice-cold methanol and separated in 1:1:1 chloroform:methanol:water. The aqueous phase containing the amino and organic acids was then dried under air at room temperature. The samples were derivatized for gas chromatography-mass spectrometry (GC-MS) analysis by adding 50 μL of 2% methoxyamine hydrogen chloride in pyridine (ThermoFisher Scientific) to each sample followed by sonication for 30 min and incubation at 40°C for 90 min. After a brief centrifugation, 70 μL of MTBSTFA +1% TBDMCS (Regis Technologies, Inc., IL, USA) was added to each sample and incubated at 70°C for 30 min to complete the derivatization. The derivatized samples were centrifuged at 17,000 × *g* for 5 min at room temperature, and then 70 μL of clear supernatant from each sample was transferred into a GC vial with a polypropylene insert. Derivatized samples were analyzed using an Agilent 7890A GC connected to an Agilent 5977B MS (33).

### Statistical analysis

All statistics were performed using GraphPad Prism 6.0. Data were analyzed using a two-tailed *t*-test. Significance was set at *p* < 0.05 after correction for multiple comparisons.

## Results

### In the failing RV, excess glucose has multiple fates including glycogen and lactate

We have previously published failing and non-failing RVs from a mouse model of heritable PAH in which BMPR2 mutation is universally expressed (27). In this model, we used a multi-omics approach incorporating metabolomics and transcriptomics to understand how multiple metabolic pathways are altered in the failing RV and confirmed data in tissue or cell culture assays as appropriate.

**FIGURE 1:**
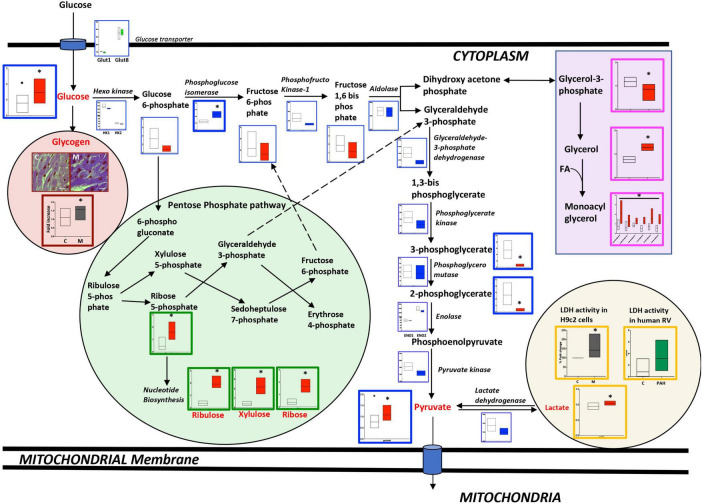
Metabolomic and transcriptomic profiling of metabolites and genes involved in the glycolysis, glycogen, pentose phosphate, and glycerol pathways in mouse RV tissue from mice with BMPR2 mutation and littermate controls. Metabolites: Control mice are in open bars (*n* = 7) and BMPR2 mutant mice (*n* = 8) are in red bars. Expression of genes: Control mice are in open bars (*n* = 3) and BMPR2 mutant mice (*n* = 3) are in blue bars. Expression of transporter genes: Control mice are in open bars (*n* = 3) and BMPR2 mutant mice (*n* = 3) are in green bars. The glycolysis pathway metabolites and genes are denoted by a blue box. Pentose phosphate pathway metabolites are denoted by a green box. Glycogen is denoted brown box. Glycerol pathway metabolites are denoted by a pink box and lactate by an orange box. LDH activity in: (1) H9c2 cells: C (control); open bar (*n* = 6) and M (BMPR2 mutant cells); gray bar (*n* = 6); and (2) human RV: C; open bar (*n* = 4) and PAH; green bar (*n* = 5). In RV cardiomyocytes from control and BMPR2 mutant mice, glycogen staining was bluish-purple in color (Magnification 100X and scale bar 10 um) and analyzed using Image J. C (control mice) open bar (*n* = 6), M (BMPR2 mutant mice) gray bar (*n* = 6). The metabolites, gene expression, glycogen localization, and LDH activity data are represented by a floating bar (min to max) with line at mean. Floating bars include all the points from min to max with line at mean, except for the metabolites glucose and pyruvate where the outliner is denoted by a black circle in control mice. *p < 0.05 was considered statistically significant using an unpaired *t*-test.

The metabolomic analysis in failing RV vs. non-failing RV (as shown in Figure 1 and Supplementary Tables 2, 3), demonstrates that glycolysis pathway metabolites; glucose (*p* < 0.01), pyruvate (end metabolite of glycolysis) (*p* < 0.02), and lactate (*p* < 0.01) were significantly increased in the failing RV, however, fructose 1,6-biphosphate and 2 and 3-phosphoglycerate (intermediate metabolites of glycolysis pathway) (*p* < 0.01) were significantly decreased. In the failing RV, in addition to an increase in glucose and pyruvate (1) intermediates of the pentose phosphate pathway (PPP) which include ribose 5-phosphate, ribulose, xylulose, and ribose (metabolites involved in nucleotide biosynthesis), (2) monoacylglycerol and glycerol (intermediates involved in triglyceride synthesis) and glycogen (the stored form of glucose) were also significantly increased (*p* < 0.05). We confirmed excess glycogen in the failing RV by PAS staining (*p* < 0.05). Corresponding epigenomic data using NanoString transcriptomic analysis indicated that in the failing RV, expression of the glycolysis pathway enzyme genes was either reduced (phosphofructokinase-1 and GAPDH) (*p* < 0.05) or trended lower compared to non-failing RV, except for the expression of phospho-glucose isomerase gene, which demonstrated a significant increase (*p* < 0.05). Since metabolomics data indicated an increase in pyruvate as well as lactate in failing RV with BMPR2 mutation, we tested the activity of lactate dehydrogenase in H9c2 cells with and without BMPR2 mutation and confirmed in RV tissue from donors and PAH patients. There was a significant increase in lactate dehydrogenase enzyme activity (*p* < 0.02) in mutant cells compared to control cells. While the differences in human RV tissue were not statistically significant, the trend was in the same direction (*p* = 0.1). Taken together, these data suggest that in the failing RV, excess glucose can be converted to lactate, and further it may also be utilized for glycogen synthesis as well as for glycerol and nucleic acid synthesis. Further, our data suggests that the metabolomic differences may not be due to alterations at the transcriptomic level in most cases.

**FIGURE 2:**
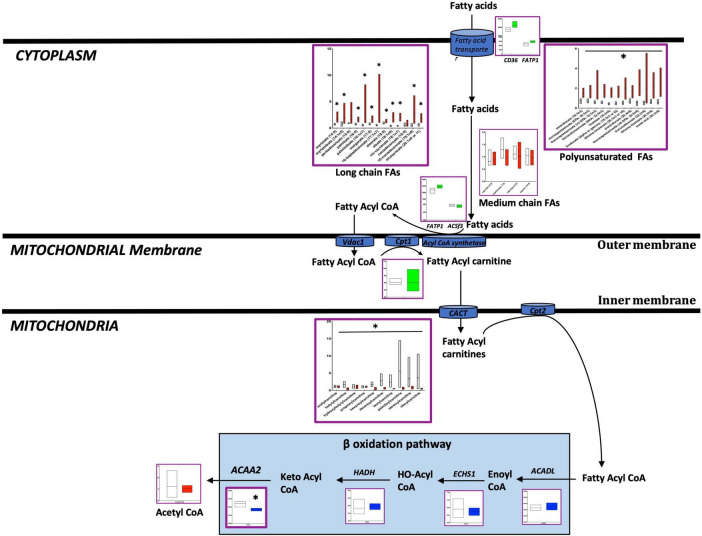
Metabolomic and transcriptomic profiling of metabolites and genes involved in the fatty acid pathway in mouse RV tissue from BMPR2 mutant mice and littermate controls. Metabolites: Control mice are in open bars (*n* = 7) and BMPR2 mutant mice (*n* = 8) are in red bars. Expression of genes: Control mice are in open bars (*n* = 3) and BMPR2 mutant mice (*n* = 3) are in blue bars. Expression of transporter genes: Control mice (*n* = 3) are in open bars and BMPR2 mutant mice (*n* = 3) are in green bars. The fatty acid pathway metabolites and genes are denoted by a purple box. The metabolites and genes involved in ß oxidation pathway are denoted by the solid light blue rectangle. The metabolites and gene expression data are represented by a floating bar (min to max) with line at mean. Floating bars include all the points from min to max with line at mean. *p < 0.05 was considered statistically significant using an unpaired *t*-test.

In the failing RV, a reduction in the transfer of LCFAs to mitochondria may underlie the accumulation of LCFAs in the cytoplasm: We and others have previously shown increased fatty acid uptake and accumulation in PAH-RV (27). Here, we compared metabolomic differences in the failing and non-failing RV (Figure 2 and Supplementary Tables 2, 3). We found that long chain fatty acids (LCFAs) (27) and polyunsaturated FAs were significantly increased (*p* < 0.05) whereas fatty acyl-carnitines (LCFAs are transferred to mitochondria for β-oxidation as fatty acylcarnitines) (27) were significantly decreased (*p* < 0.05) in the failing RV. Acetyl-CoA, an end metabolite of β-oxidation and a key metabolite entering the TCA cycle, however, remained unchanged. Corresponding transcriptomic data indicated that in the failing RV, the expression of the ACAA2 gene (a final enzyme involved in mitochondrial β-oxidation) was significantly decreased (*p* < 0.05), however, the expression of other β-oxidation pathway enzyme genes (ACADL, ECHS1, HADH) remained unchanged. In addition, fatty acid transporter genes which include (1) transport of fatty acids from outside to cytoplasm (CD36 and FATP1) and (2) transport of fatty acids from the cytoplasm to mitochondria (FAPT1 ACSf3 and CPT1) were unchanged in failing RV. This data suggests that in the failing RV, a reduction in the transfer of LCFAs to mitochondria could result in the accumulation of LCFAs in the cytoplasm. While there was reduced ACAA2 expression, the metabolites before and after were unaffected, suggesting that beta oxidation enzymatic expression and function may be intact in failing vs. non-failing PAH RVs.

**FIGURE 3:**
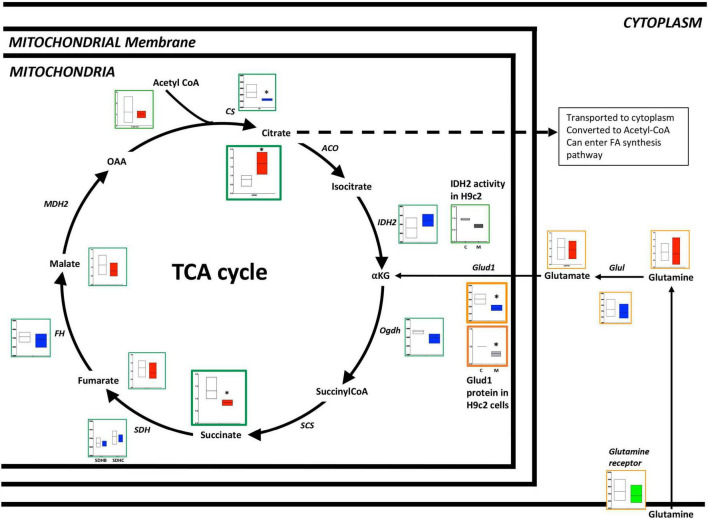
Metabolomic and transcriptomics profiling of metabolites and genes involved in the TCA cycle and glutamine pathways in mouse RV tissue from BMPR2 mutant mice and littermate controls. Metabolites: Control mice are in open bars (*n* = 7) and BMPR2 mutant mice (*n* = 8) are in red bars. Expression of genes: Control mice are in open bars (*n* = 3) and BMPR2 mutant mice (*n* = 3) are in blue bars. Expression of transporter genes: Control mice (*n* = 3) are in open bars and BMPR2 mutant mice (*n* = 3) are in green bars. The TCA cycle metabolites and genes are denoted by a green box. Glutamine pathway metabolites are denoted by an orange box. IDH2 activity in. H9c2 cells: C (open bar) *n* = 2 and M (blue bar) *n* = 2 and Glud1 protein in H9c2 cells: C (open bar) *n* = 4 and M (gray bar) *n* = 4. The metabolites and gene expression data are represented by a floating bar (min to max) with line at mean. Floating bars include all the points from min to max with line at mean. *p < 0.05 was considered statistically significant using an unpaired *t*-test.

In the failing RV, the TCA cycle is altered at multiple checkpoints with excess accumulation of citrate: There is prior work demonstrating impairment of the TCA cycle in PAH (34, 35). When we performed a comprehensive metabolomic analysis of failing vs. non-failing RVs in a mouse model (Figure 3 and Supplementary Tables 2, 3), we observed that citrate (a key intermediate of the TCA cycle) was increased (*p* < 0.001) whereas succinate (TCA cycle intermediate, downstream of citrate) was decreased (*p* < 0.01) in the failing RV, however, other metabolites of the TCA cycle remained unchanged. Corresponding transcriptomic data indicated that in the failing RV, the CS gene was significantly reduced (*p* < 0.05), while other genes involved in the TCA cycle (IDH2, SDH, and FH) either trended lower or remained unchanged. We further tested IDH enzyme activity (one of the key enzymes of the TCA cycle involved in the conversion of iso-citrate to α-ketoglutarate) in H9c2 cells with and without BMPR2 mutation. Compared to control cells, there was a significant decrease in IDH activity (*p* < 0.02) in BMPR2 mutant cells. Taken together, this data indicated that in failing RV, the TCA cycle is altered at multiple checkpoints with excess accumulation of citrate, likely at least in part due to decreased IDH activity. Excess citrate may be used in FA synthesis.

In addition to fatty acids and glucose as a source of carbon, glutamine can enter the TCA cycle and be utilized as a source of carbon for energy production (36, 37). When we performed metabolomic analysis of failing vs. non-failing RV in the mouse model of PH, we observed no difference in the levels of metabolites glutamine and glutamate (Figure 3). Corresponding epigenomic data using transcriptomics indicated that although the expression of glutamine receptor and Glu1 remained unchanged, the expression of the Glud1 gene was significantly decreased (*p* < 0.05) (Figure 3). In H9c2 cells, our results indicated that Glud1 protein expression was significantly reduced in BMPR2 mutant cells (*p* < 0.01) compared to controls. This data indicates that the capacity to utilize glutamine as a carbon source for the TCA cycle is not upregulated in the failing RV.

### Cultured cardiomyocytes with BMPR2 mutation do not show an increase in lactate uptake

**FIGURE 4:**
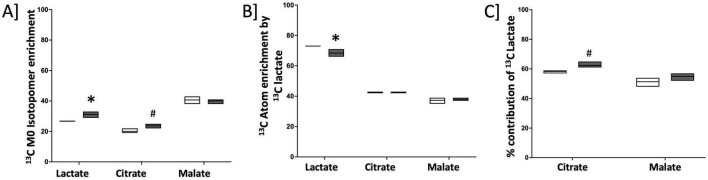
Endogenous lactate pool is increased in cultured cardiomyocytes with mutated BMPR2: **(A)** ^13^C M0 Isotopomer enrichment in lactate and TCA intermediates. **(B)** ^13^C Atom enrichment by ^13^C lactate in lactate and TCA intermediates. **(C)** % contribution of total ^13^C lactate in TCA cycle intermediates. C; control cells: black circles (*n* = 3), M; BMPR2 mutant cells: red circles (*n* = 3). Data is represented as a scatter dot plot with median, *n* = 3 control and BMPR2 mutant H9c2 cells. *p < 0.05 was considered statistically significant and #*p* = 0.05 using an unpaired *t*-test.

We next sought to determine whether increased lactate in RV cardiomyocytes is due to the increased uptake of lactate from circulation. It is known that lactate can be taken up by the cells and used as a source of carbon by converting to pyruvate which then enters the TCA cycle. To assess whether circulating lactate is taken up by cardiomyocytes with BMPR2 mutation, we cultured control and BMPR2 mutant cells in the presence of 10 mM sodium [U-^13^C_3_]L-lactate (a stable isotope of lactate in which all 3 carbon atoms are substituted with carbon-13 that is easily detected by mass spectroscopy) + 2 mM glucose for 24 hrs. Our results indicated that, compared to control cells, mutant cells exhibited a modest but significant increase (*p* < 0.02) in endogenous (M0) lactate (Figure 4A) and a significant decrease (*p* < 0.03) in the ^13^C-lactate intracellular pool (taken up from cell culture media) (Figure 4B). Next, we analyzed the incorporation of ^13^C-lactate in the TCA cycle intermediates (Figures 4A, B). Our data indicated a trend toward an increase in the relative contribution of ^13^C-lactate to the intracellular citrate pool but not to the malate pool (Figure 4C). Further, our data suggested that the trend toward an increase (*p* < 0.06) in the citrate pool is driven by endogenous lactate (Figures 4A, B) which did not propagate to malate. Taken together, these cell culture studies confirm our findings in the failing RV (in the animal model of PH with BMPR2 mutation) and suggest that BMPR2 mutant RV cardiomyocytes show an increase in lactate possibly due to an increase in the activity of lactate dehydrogenase and can potentially contribute to increased lactate in circulation.

**FIGURE 5:**
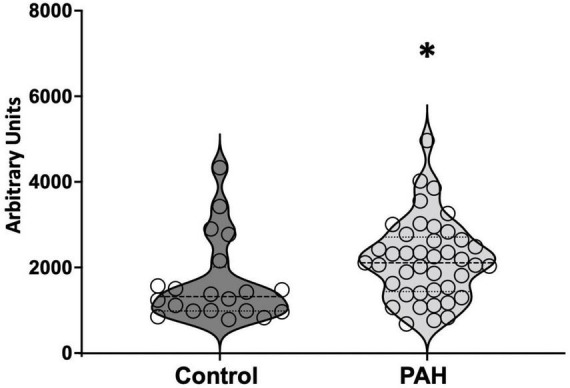
Lactic acid measured in circulation in control and PAH patients. The data is represented by a violin plot showing all the points. *p < 0.05 was considered statistically significant using an unpaired *t*-test.

In PAH patients with BMPR2 mutation, genes and metabolites involved in glycolysis, fatty acid pathways, and the TCA cycle are significantly increased in circulation: We sought to understand how these metabolic changes identified in the failing RV were reflected in the plasma. In PAH patients compared to healthy controls, plasma acyl-carnitines (*p* < 0.03) (Table 1) and lactic acid (*p* < 0.02) (Figure 5) were significantly increased, glutamate trended higher and remaining metabolites remained unchanged. Corresponding epigenomic data using transcriptomics in the plasma showed that the expression of enzymes involved in the glycolysis pathway (HK1, HK2, HK3, PFKM, G3PDH, PGK1, ENO1, PKM, LDHA), fatty acid oxidation pathway (CD36), TCA cycle (SDHC) and glutamine pathway (Glud1) were significantly increased in PAH patients (Table 2). All the other genes involved in these pathways also trended higher in PAH patients (Table 2). Taken together, this data indicates that in PAH patients, lactate and long-chain acylcarnitines are increased in circulation and increased lactate in circulation mirrors the findings in failing mouse RV. However, unlike in failing mouse RV, expression of genes involved in the glycolysis pathway, FA pathway, TCA cycle, and glutamine pathway are increased in PAH in circulation suggesting an origin of these findings to be other than RV tissue.

**TABLE 1:** Glycolysis, fatty acid, TCA cycle, and glutaminolysis pathway metabolites identified in human plasma.

	Control		HPAH		
		SEM		SEM	*P*-value
**Glycolysis pathway**					
Glycolysis metabolites	702.1	26.1	648.3	37.3823	0.5
**FA pathway**					
C16 acylcarnitines	0.082	**0.004**	**0.116**	**13.000**	**0.01**
C18 acylcarnitines	0.029	0.002	0.036	0.004	0.1
C18:l acylcarnitines	**0.097**	**0.006**	**0.162**	**0.031**	**0.002**
C18:2 acylcarnitines	**0.051**	**0.003**	**0.103**	**0.023**	**0.001**
**TCA cycle and glutaminolysis pathway**					
Citrate	29.5	1.3	34.9	4.8	0.17
Glutamate	50.2	6.7	86.3	16.5	0.07
Glutamine	549.7	15.2	550.1	40.4	1.00
Glutaminolysis metabolites	0.72	0.05	0.74	0.10	0.9

Statistical analysis was performed using multiple *t*-tests and *p* < 0.05 was considered significant. The unit of measurement for all the metabolites is denoted as “Scaled intensity.” The values in bold represent that the *p*-value is significant.

**TABLE 2:** Expression of genes involved in glycolysis, fatty acid, TCA cycle and glutaminolysis pathway in human plasma.

	Control		PAH		
		SEM		SEM	*P*-value
**Glycolysis pathway genes**					
GLUT1	**26.6**	**2.9**	**49.3**	**6.6**	**0.02**
GLUT14	1,416.5	193.6	2,604.5	839.0	0.23
HK1	**465.5**	**29.8**	**794.5**	**131.9**	**0.01**
HK2	**360.7**	**44.7**	**711.8**	**103.8**	**0.01**
HK3	**115.5**	**14.7**	**209.5**	**44.3**	**0.05**
GPI	156.8	12.5	201.5	29.8	0.21
PFKM	**46.9**	**3.9**	**75.3**	**14.8**	**0.03**
GAPDH	**5,971.9**	**398.2**	**9,427.0**	**427.8**	**0.003**
PGK1	**2,612.5**	**161.0**	**3,904.5**	**206.3**	**0.01**
ENO1	**462.5**	**32.5**	**719.8**	**88.7**	**0.003**
PKM	**614.5**	**38.2**	**978.5**	**128.0**	**0.02**
LDHA	**830.1**	**59.8**	**1,758.5**	**264.9**	**0.001**
LDHB	3,948.7	375.8	7,166.0	2,765.3	0.57
**FA pathway genes**					
CD36	**917.0**	**80.5**	**1,850.0**	**181.6**	**0.001**
ACSF3	37.4	4.4	47.3	11.4	0.39
CPT1a	118.1	15.8	193.0	50.4	0.23
ECHS1	71.3	3.5	91.8	21.7	0.85
HADH	145.3	10.2	215.0	57.5	0.48
ACAA2	205.0	12.9	294.3	54.4	0.06
ACAT1	65.5	4.2	121.3	31.2	0.08
**TCA cycle genes**					
CS	345.7	25.7	465.3	97.2	0.28
IDH2	493.3	38.7	603.0	103.8	0.51
OGDH	528.9	39.6	740.0	127.5	0.18
SDHB	339.0	29.7	504.3	92.7	0.07
SDHC	**545.5**	**33.3**	**843.3**	**59.6**	**0.003**
FH	201.4	14.6	317.5	70.7	0.18
GLUD1	**556.4**	**35.9**	**1,049.0**	**204.7**	**0.003**

Statistical analysis was performed using multiple *t*-tests and *p* < 0.05 was considered significant. The unit of measurement for all the genes is denoted as “copy number.” The values in bold represent that the *p*-value is significant.

## Discussion

In PAH, metabolic abnormalities in multiple pathways are well-recognized features of RV dysfunction. We have sought a novel mechanistic approach to understanding the key metabolic mechanism of RV failure by integrating metabolomic and epigenomic data using transcriptomics in failing RV and non-failing RVs using a mouse model of RV failure with BMPR2 mutation and correlating the findings with plasma from PAH patients. By layering metabolomics and transcriptomics data, we were able to show that in the failing RV, multiple metabolic pathways are altered as measured by metabolite-enzyme gene expression/activity and we were able to identify where the breakpoints exist. In this study, we also learned that derangement of metabolite-enzyme gene expression is mostly tissue-specific, however, we found that lactate, one of the metabolites, that was increased both in RV tissue and circulation has the potential to be a mechanistic biomarker of RV dysfunction.

In the past, a multi-omics approach (integrating transcriptomics with proteomics) was used in cellular models such as pulmonary vascular endothelial cells from healthy donors to understand the role of sexual dimorphism in PH (38), and, in normal pulmonary artery smooth muscle cells to understand the crosstalk between PDGF signaling and BMPR2/SMADs axis (39). Similarly, in sugen hypoxia animal model PH, a multi-omics approach (integrating transcriptomics and metabolomics) was used to study the effect of Chrysin, a photochemical with pharmacological activity, on gene expression and levels of mitochondrial metabolites in the right ventricle (8). On the other hand, single-omics studies such as metabolomic studies were conducted in multiple organs in the murine model of PAH, to assess the alterations in metabolic programming associated with PAH that precedes phenotypic changes (37, 40). A targeted and untargeted metabolomics approach was also used to study the relationship between intracellular metabolic abnormalities and the serum metabolome in the Sugen hypoxia model of PH (RV and serum), isolated cultured pulmonary endothelial cells, and serum collected from PAH patients (41). A stable isotope metabolomics analysis of pulmonary artery smooth muscle and endothelial cells was done to show substrate preference in response to TGF-ß (16). Similarly, using single-cell RNA sequencing of lung endothelial cells from sugen hypoxia, and a monocrotaline rodent model of PH, differential gene expression, gene set enrichment, cell–cell communication, and trajectory reconstruction was done and its relevance to human PAH was assessed in multiple independent blood and lung transcriptomic data sets (9). Taken together, in the past, most of the PAH studies were either conducted using a multi-omics approach in one tissue or a single-omics approach in multiple tissues. In this manuscript, firstly, using a multi-omics approach (integrating metabolomics and transcriptomics), we performed a detailed pathway analysis for major metabolic pathways utilizing multiple substrates (important for energy production) in normal and failing RV with mutated BMPR2. And, secondly, using a multi-omics approach (integrating metabolomics and transcriptomics), we studied the relevance of alterations in these metabolic pathways in RV failing to metabolites /gene expression in circulation in human PAH.

In our mouse model of RV failure with BMPR2 mutation, an increase in glucose metabolites (pyruvate and lactate) observed in the RV is in concurrence with studies done in PAH patients that showed increased ^18^F-fluorodeoxyglucose uptake in the right ventricle quantitatively measured by using positron emission tomography (FDG-PET) compared to healthy individuals (42–44). Metabolic profiling of RV in MCT induced rat model of PH also indicated an increase in glucose, which is consistent with our findings (45). Glycolysis generates pyruvate and if this pyruvate is not utilized to fuel oxidative phosphorylation, it is utilized for lactate production (17). In our mouse model of PH with BMPR2 mutation, we have shown an increase in lactate production which is consistent with findings in the sugen hypoxia mouse model of PH indicating an increase in lactate production in RV, but a decrease in glucose (37). In contrast to our findings, metabolic profiling of RV in sugen hypoxia or hypoxia-induced rat model of PH did not show any changes in glucose or lactate (46). Next, in our mouse model of RV failure, we have shown for the first time an increase in glycogen content in RV cardiomyocytes. Although there is no direct evidence of glycogen excess in pathology samples from human PAH patients, there is an association shown between PAH and glycogen storage disease (47). Similar to cancer cells, in PAH, increased glucose in tissue can be utilized by other biosynthetic pathways such as PPP (48). PPP generates reduced nicotinamide adenine dinucleotide phosphate (NADPH) and ribose-5-phosphate to preserve nucleotide synthesis and redox homeostasis. An increase in PPP flux in PAH patients and multiple animal models has been identified and shown to be associated with the metabolic changes that precede the development of PAH (49, 50). Our data in a mouse model of failing RV supports this notion. Further, in our mouse model of RV failure, a decrease in glycerol-3-phosphate is consistent with metabolic profiling of RV in sugen hypoxia-induced rat model of PH (45), and an increase in glycerol and monoacylglycerol suggests that these metabolites can be utilized for triglyceride synthesis or released in circulation as seen in CTEPH patients (51).

In our animal model of RV failure, HK (1 and 2) as well as glucose transporter (Glu1 and Glut8) gene expression remained unchanged which is in contrast to our previously published (52) gene expression data from human RV biopsies from PAH patients which demonstrated an increase in HK2 and glucose transporter (Glut3). As for LDHA gene expression, the MCT-induced PH rat model indicated an increase in LDH-A mRNA and protein gene (20), but, we did not observe any increase in the expression of this gene, however, we were able to demonstrate an increase in LDH activity in cultured cardiomyocytes with BMPR2 mutation and a trend toward an increase in LDH activity in human RV. IPAH patients have shown increased levels of serum lactate dehydrogenase which correlates with the disease severity and patient mortality (53).

It is well established that RV lipid accumulation is associated with right ventricular dysfunction and failure (52, 54). In PAH patients and in the mutant BMPR2 mouse model of RV failure we have shown increased RV lipid accumulation in the form of triglycerides, diacylglycerols, and ceramides, as well as long-chain fatty acids (27, 52, 54), and a decrease in fatty acyl carnitines (27). In addition to this, in this study, we show an increase in polyunsaturated fatty acids but not in medium-chain fatty acids. Our findings showing a decrease in acylcarnitines in RV is consistent with metabolic profiling findings in RV of MCT and sugen hypoxia induced rat model of PH (45) but in contrast to the sugen hypoxia and hypoxia mouse models of PH which indicated a decrease in fatty acids (37), although, this study did not specify the type of fatty acids which may be relevant given our findings are specific to long chain fatty acids.

In PAH, excess cellular lipid uptake in cardiomyocytes is mediated by CD36 (fatty acid transporter), resulting in lipid accumulation in RV (27, 52, 55). Our data indicated a trend toward an increase in CD36 and FATP1 gene expression in RV in our mouse model of PH with BMPR2 mutation. In the MCT-induced rat model of PH, CD36 mRNA, and protein expression were shown to increase in hearts (but were not studied specifically in RV) (20). Once taken up, fatty acids are converted to fatty acyl CoA and transported across the mitochondrial membrane as acyl-carnitines and converted back to fatty acyl CoA to undergo mitochondrial β-oxidation. A series of transporter proteins are involved in this process. Carnitine palmitoyltransferase 1 (CPT1) is the rate-limiting gene while other transport molecules assist with the fatty acid transport across the mitochondrial membranes. In our mouse model, although the expression of FATP1, ACSf3, and CPT1 genes remained unchanged in RV, we have shown a significant decrease in fatty acylcarnitines (27). Further in BMPR2 mutant cardiomyocytes, we have shown impaired mitochondrial function (27, 52, 54). However, we believe that disruption of the mitochondrial β-oxidation may not be at the transcriptional level, because at the transcriptional level other than significant reduction in the ACAA2 gene expression, mitochondrial β-oxidation genes are unchanged suggesting that impaired mitochondrial function is metabolically regulated.

Prior published papers suggest a metabolic switch from OXPHOS energy production to glycolysis in a dysfunctional PAH RV (19). Our data indicates that in the failing RV, energy metabolism is altered not just due to a reduction in glucose oxidation but also because of disruption in the TCA cycle (as indicated by our metabolomics data). Unlike metabolic profiling findings in RV of sugen hypoxia induced rat model PH (45), our data demonstrates an increase in citrate but decrease in succinate (a metabolite downstream of citrate in TCA cycle), suggesting a break in TCA cycle. Further, IDH2 enzyme activity was also significantly decreased in a BMPR2 mutation-specific manner. We, therefore, hypothesize that the decrease in TCA cycle activity is not just due to incomplete mitochondrial β-oxidation and response to the metabolic switch from glucose and lipid oxidation toward aerobic glycolysis but also due to breaks in the TCA cycle itself. Glutaminolysis has been shown to play an important role in RV hypertrophy and RV failure, in PAH and MCT animal models of PH, (56), however, in our model of RV failure, our metabolomic and transcriptomic data indicate that the capacity to oxidize glutamine is not elevated in BMPR2 mutated RV cardiomyocytes.

PAH patients with systemic sclerosis have shown an increase in lactate (3), which supports our data where we have also shown increased lactate in circulation in the PAH patient population. Increased lactate is shown to be one of the predictors of mortality in patients with connective tissue disease—PAH with right heart failure (57) and in patients with heart failure elevated levels of lactate are shown to relate to poor clinical outcomes (58–60). Similarly, animal models of PH have also shown a significant increase in glucose consumption, increased lactate production, and an increase in serum lactate (61). As a cautionary note, we should acknowledge that in PAH and animal models of PH, skeletal muscle is an important source of lactate in circulation (62) and demonstrates elevated lactate dehydrogenase activity (63). In pulmonary vascular disease, glycolytic reprogramming has been shown to contribute to the production of phospholipids, nucleotides, and amino acids required to sustain cellular replication (64, 65), which is similar to our findings in RV failure in an animal model.

Our study indicates that in the failing RV with BMPR2 mutation, there are several impacted metabolic pathways that include glycolysis, the TCA cycle and fatty acid metabolism and these are primarily altered at the level of metabolome and not transcriptome. Through comparison with human plasma, our data further suggests that these changes are likely tissue-specific and that plasma may not fully reflect these relevant tissue-specific changes. Thus, although lactate can potentially serve as a marker of RV dysfunction in PAH, increased lactate in circulation may not just be because of increased lactate production in the PAH-RV and warrants further investigation. We, therefore, conclude that metabolites in circulation have a more complex relationship with deranged metabolic pathway metabolites in tissues and the correlation between plasma and tissue of interest would be highly impactful to understand in what ways plasma does and does not reflect RV pathobiology.

## Author contribuitons

AH: Conceptualization, Funding acquisition, Resources, Supervision, Validation, Writing – review & editing. NF: Data curation, Methodology, Writing – review & editing. KS: Data curation, Methodology, Writing – review & editing. IT: Data curation, Methodology, Writing – review & editing. SS: Methodology, Writing – review & editing. EA: Methodology, Resources, Writing – review & editing. JY: Conceptualization, Formal analysis, Resources, Writing – review & editing. EB: Resources, Writing – review & editing. JW: Methodology, Resources, Supervision, Writing – review & editing. MT: Conceptualization, Data curation, Formal analysis, Methodology, Resources, Validation, Visualization, Writing – original draft, Writing – review & editing.

## Data Availability

The original contributions presented in the study are included in the article/Supplementary material, further inquiries can be directed to the corresponding author/s.
