# Supplementary material for: A multimodal approach identifies lactate as a central feature of right ventricular failure that is detectable in human plasma
Source: Front Med (Lausanne). 2024 Sep 12;11:1387195. doi: 10.3389/fmed.2024.1387195 (PMC11428650; doi:10.3389/fmed.2024.1387195)
Supplement: Supplementary file 1 [file Table_1.DOCX]

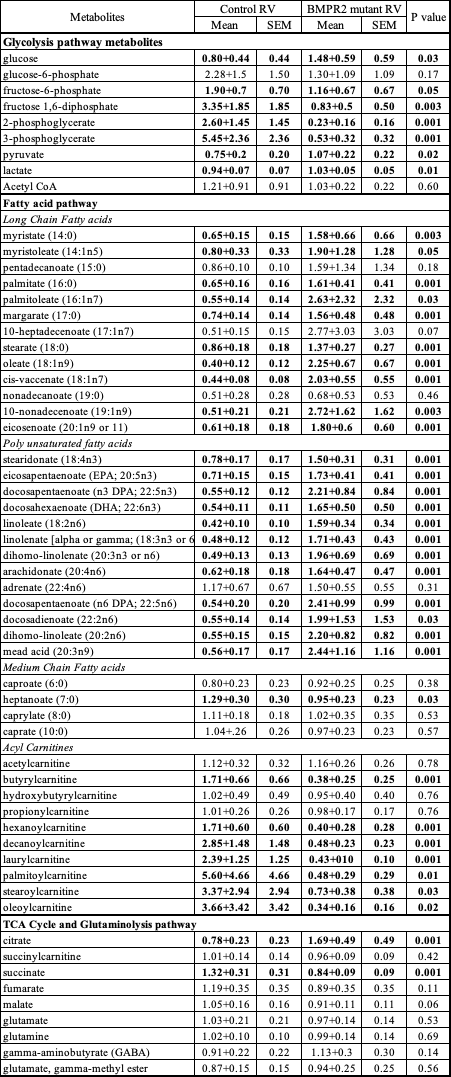


**Supplementary Table 1: Glycolysis, fatty acid, TCA cycle, and glutaminolysis pathway metabolites identified in failing mouse RV with mutated BMPR2) vs nonfailing mouse RV tissue.**
