# Supplementary material for: A multimodal approach identifies lactate as a central feature of right ventricular failure that is detectable in human plasma
Source: Front Med (Lausanne). 2024 Sep 12;11:1387195. doi: 10.3389/fmed.2024.1387195 (PMC11428650; doi:10.3389/fmed.2024.1387195)
Supplement: Supplementary file 3 [file Table_3.DOCX]

ACE2

FOXO3

BMPR2

ID1

FGF10

FGF2

SOX17

TBX4

CYP19A1

ESR1

ESR2

PGR

SHBG

SERPINE1

NPR3

PTGIR

PTGIS

PGFS

PTGDR

PTGDS

PTGFR

TBXA2R

ACSS2

ATP5F1A

GLUT4

HMGCS2

PMM1

Corin

CXCL12

Leptin

**Supplementary Table 3: C**ustom panel_C7746 (Pulmonary **A**rterial **H**ypertension related genes)
